# Vibrational Spectroscopy for Identification of Metabolites in Biologic Samples

**DOI:** 10.3390/molecules25204725

**Published:** 2020-10-15

**Authors:** Kevin V. Hackshaw, Joseph S. Miller, Didem P. Aykas, Luis Rodriguez-Saona

**Affiliations:** 1 Department of Internal Medicine, Division of Rheumatology, Dell Medical School, The University of Texas, 1601 Trinity St, Austin, TX 78712, USA; 2 Department of Medicine, Ohio University Heritage College of Osteopathic Medicine, Dublin, OH 43016, USA; Mille718@miamioh.edu; 3 Department of Food Science and Technology, Ohio State University, Columbus, OH 43210, USA; Perenaykas@gmail.com (D.P.A.); rodriguez-saona.1@osu.edu (L.R.-S.); 4 Department of Food Engineering, Faculty of Engineering, Adnan Menderes University, Aydin 09100, Turkey

**Keywords:** vibrational spectroscopy, Raman spectroscopy, infrared spectroscopy, fingerprinting, metabolites, spectroscopy, diagnostics, biofluid, biomarker

## Abstract

Vibrational spectroscopy (mid-infrared (IR) and Raman) and its fingerprinting capabilities offer rapid, high-throughput, and non-destructive analysis of a wide range of sample types producing a characteristic chemical “fingerprint” with a unique signature profile. Nuclear magnetic resonance (NMR) spectroscopy and an array of mass spectrometry (MS) techniques provide selectivity and specificity for screening metabolites, but demand costly instrumentation, complex sample pretreatment, are labor-intensive, require well-trained technicians to operate the instrumentation, and are less amenable for implementation in clinics. The potential for vibration spectroscopy techniques to be brought to the bedside gives hope for huge cost savings and potential revolutionary advances in diagnostics in the clinic. We discuss the utilization of current vibrational spectroscopy methodologies on biologic samples as an avenue towards rapid cost saving diagnostics.

## 1. Introduction

In the field of diagnostic medicine, mass spectroscopy (MS) and nuclear magnetic resonance (NMR) spectroscopy have found wide applications, including in the detection of infectious, cardiovascular, neurological diseases or cancer biomarkers [1]. Even though MS and NMR spectroscopy techniques provide selectivity and specificity for metabolite screening, they are labor-intensive and require costly instrumentation, complex sample pre-treatment, and well-trained technicians to operate the instrumentation, making them less amenable for implementation in clinics. Clinical medicine urgently needs a new methodology for the rapid and reproducible detection of unknown metabolites in biological samples. Vibrational spectroscopy (infrared and Raman spectroscopy) offers rapid, high-throughput, and non-destructive analysis of a wide range of samples through their unique “fingerprinting” capabilities.

The basis of vibrational spectroscopy is the transitions between quantized vibrational energy states of molecules due to the interaction between the material and the radiation from a light source [2,3]. Vibrational spectroscopy consists of infrared and Raman spectroscopy. Even though both the near-IR (12,500–4000 cm^−1^) and mid-IR (4000–400 cm^−1^) are part of the infrared spectroscopy, most medical diagnoses researchers focus on the mid-IR part of the spectrum because of the fundamental vibrations in the mid-IR region provides sharper bands and provides more information on disease diagnosis rather than the overtone and harmonic vibrations that are provided by the near-IR region [4,5]. The mid-IR spectroscopy is based on the interaction between the sample and the IR beam, which absorbs by the functional groups in the sample and vibrates as stretching, bending, deformation or combination, and provides the fingerprint characteristics of the chemical or biochemical substances in the sample [4]. A major hurdle for FT-IR spectroscopy is the interference of the water in the mid-IR region, which masks some key biochemical information, especially in the Amide I (~1650 cm^−1^) and lipids (3000–3500 cm^−1^) absorption regions, and the water absorption could inhibit the light from penetrating the sample [6,7]. There are several approaches to overcome the water problem including the removal of the pure or scaled water spectrum from the acquired spectrum, dehydrating the sample, using D_2_O solution, or lowering the effective path length significantly by using the attenuated total reflectance (ATR) as a sampling technique [4,6,7].

Raman spectroscopy is an inelastic light-scattering phenomenon, the incident photon is irradiated on the sample, and the molecules scatter the light. Although most of the scattered light has the same frequency as the incident light, some of them have different frequencies due to the interaction between the oscillation of light and molecular vibration. This phenomenon is called Raman scattering and, unlike IR spectroscopy, Raman spectroscopy has a very weak water signal and minimal water interference, which is a great advantage for the analysis of the biological samples [8]. Raman spectroscopy can offer direct measurements from the biofluids, single cells, in vitro, or even in vivo fiber-optic sampling for bladder and prostate, esophagus, and skin, cervix, and arteries [4]. Furthermore, Raman spectroscopy is a non-destructive and non-invasive (wavelength and power-dependent) technique; it requires minimal sample preparation, and simultaneous detection of macromolecules suitable for chemical analysis, quantification, classification, and the imaging of biological samples [9]. On the other hand, the Raman effect is very weak, and only 1 in 10^8^ photons undergo Raman scattering [10]—to overcome this drawback longer acquisition times could be used, which could cause damage the sample due to the laser exposure [9]. The other method to amplify the Raman spectroscopy’s inherent signal weakness is by using the surface-enhanced Raman scattering (SERS) technique. SERS uses nanoscale roughened metallic surfaces (typically gold or silver), which could greatly enhance the order of the Raman signal (~10^8^) [11]. The signal enhancement can increase even up to 10^11^ with the surface-enhanced resonance Raman spectroscopy (SERRS) [12]. Advancements in the Raman instrumentation along with the SERS phenomenon, have boosted the application of the Raman as a diagnosis tool [13]. The other hurdle for Raman spectroscopy is the fluorescence interference, which happens when visible wavelength lasers are used [14]. Especially during in vivo analysis, the fluorescence background signal can dominate the fingerprint region of the spectra [4,9]. The fluorescence interference can be removed mathematically or by illuminating the sample with the laser beam for a long time as a pre-treatment (this process is also known as “bleaching” or “photobleaching”) or by using longer wavelength lasers (i.e., 1064 nm) [4,14].

The IR spectra’s unique profile is the result of molecules absorbing IR radiation, which causes a change in the dipole moment of the molecules as a result of induced vibrational motion that rearranges their charge distribution. On the other hand, for Raman scattering to occur, the molecular polarizability in their electron cloud must change during the molecular vibration, and there is no need to change dipole moment. IR and Raman spectroscopy are complementary to each other, since the IR absorption is active for anti-symmetrical vibrations that change the dipole moment, and Raman scattering is active for the symmetric vibration that can change the polarizability [15].

**Figure 1:**
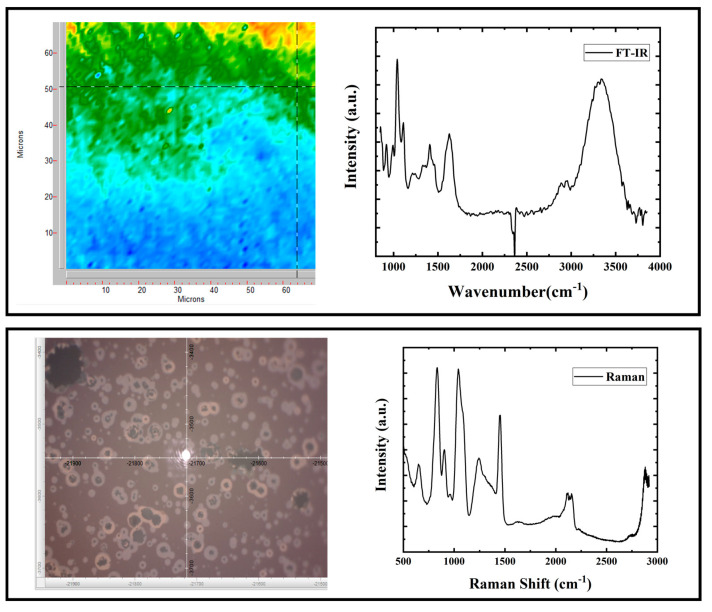
Examples of obtained spectra from Raman and FT-IR microscope imaging systems and the images of measurement locations.

FT-IR and Raman spectro-imaging systems enable spectral collection from each pixel in the image, suitable to explore the heterogeneous samples, notably the biological samples, with a resolution close to a cellular level [5,12,16]. Imaging systems combine the visual perception of digital imaging technology with the objective cellular level information from the vibrational spectroscopy [17]. Figure 1 shows an example of the FT-IR and Raman imaging systems and their corresponding spectra.

Over the last decade with rapidly evolving technology, spectroscopic tools are getting smaller and more portable, and with these advancements, spectroscopic tools are becoming more valuable for routine on-site analysis and point-of-care applications [18]. In particular, the advancements in the production of micro-electro-mechanical systems and high-performing chip technology lead the bench-top vibrational spectrometers to be miniaturized into commercial portable and handheld Raman and infrared spectrometers, by keeping the analytical precision and the spectral resolution equivalent to bench-top equivalents. Furthermore, the miniaturization reached a point, where the spectrometers can be attached to smartphones for medical diagnosis and clinical assays without going to a laboratory, especially in remote and low-resource areas [19].

Biologic samples, when easily accessible, particularly biofluids, make an attractive target for tools of vibrational spectroscopy. Tissue samples excised or retrieved from areas of the disease can be quickly examined through spectroscopic means. Major inroads in this regard have been seen in various forms of cancer. Biofluids are typically readily accessible with low to minimal levels of invasiveness needed to obtain the sample. These fluids are attractive due to the metabolites that they may procure as a result of being obtained from the surrounding milieu of the area or site of interest. From the initial literature review until August 2020, PubMed, Embase, Google Scholar, and Scopus were searched for applicable studies. The overview of clinical studies performed by using Raman and FT-IR spectroscopy units as a diagnosis tool in vivo in humans are given in Table 1 and Table 2, respectively. The brief overview of the analytes detected from biofluids using Raman and FT-IR and their detection limits are given in Table 3. In each instance of our literature review, the search terms utilized were the biofluid or tissue of interest coupled with IR, Raman, or vibrational spectroscopy.

**Figure 2:**
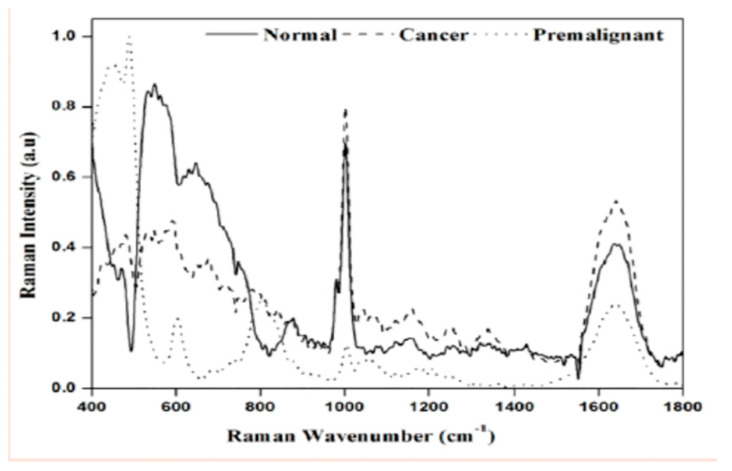
Raman spectroscopic intensity variations and peak shift among urine samples of three groups [85].

Urine: Owing to urine’s composition of substances cleared through blood filtration and function in waste storage and clearing of waste, it may be a promising biofluid for early identification of biomarkers associated with disease states or early damage associated with various diseases [82,83]. Furthermore, collection of urine can be noninvasive, performed discreetly at the patient’s bedside, and obtained in large quantities compared to blood and other biofluids. Historically, analysis of urine has enabled the diagnosis of various diseases of the kidneys and glomerulus [83]; however, recent studies suggest that urine is a promising biofluid for the early diagnosis of bladder cancer, prostate cancer, and type 2 diabetes [83,84,85]. Jaychandran and colleagues showed identification of key urine metabolites with Raman peaks observed for uric acid (567 cm^−1^), FAD (606 cm^−1^), creatinine (692 cm^−1^ in normal, 1336 and 1427 cm^−1^ in malignant), ethanol peak (890 cm^−1^), urea (1002 cm^−1^glucose peak (1046 cm^−1^), tryptophan (1417 cm^−1^ in normal and 1547 cm^−1^ in premalignant and malignant), indoxylsulphate (1615 cm^−1^ in normal, 1351 cm^−1^ in malignant) (Figure 2). Recent research has associated elevated concentrations of various branched chain amino acids in urine samples to insulin resistant and diabetic patients [86]. The use of various vibrational spectroscopy techniques in the analysis of urine for early assessment and diagnosis is supported by the above-mentioned research. In fact, vibration spectroscopy techniques may elucidate further biomarkers in urine for various disease states and improve diagnostic techniques for physicians and healthcare specialists. Specifically, this technique allows for quick, highly specific, highly selective, and non-invasive sample characterization [87].

**Figure 3:**
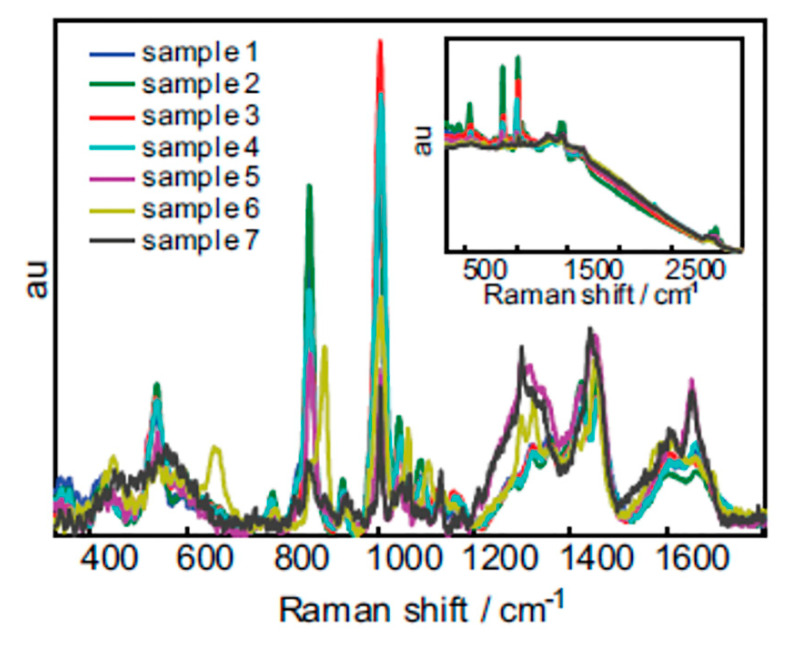
Average raw (insert) and after baseline correction spectra from seven sweat samples [93].

Sweat: Despite sweat gland’s function as a secretory and, more specifically, excretory organ for bio-waste, the clinical use of sweat as a biofluid is scarce and necessitates further study [88]. Previous research has identified various metabolites in sweat and recent advancements allow for the reliable and rapid collection of this biofluid. Therefore, sweat may be a promising fluid for identifying unknown metabolites and may allow for rapid clinical diagnoses of various disease states using vibrational spectroscopy [88,89]. In fact, the use of sweat in the diagnosis of cystic fibrosis has been well documented and its use as a diagnostic biofluid in other diseases, such as Addison’s disease, Parkinson’s disease, and schizophrenia has been inferred [88,90]. Raman spectroscopy has identified significant variance among unique sweat samples resulting from amino acids, proteins, and metabolites in the samples [91]. Figure 3 reflects the slight variation from seven different sweat samples. Visually, these spectra are all similar, although some variations are evident, including the variation of the fluorescence background (Figure 3). Surface enhanced Raman spectroscopy (SERS) using silver nanoparticles (AgNPs) was used to analyze sweat left by a fingerprint [92]. Owing to high sensitivity of SERS, the team identified biomolecules with extremely small concentrations. Based on previous results, sweat contains metabolites that can be used for the early diagnosis of various diseases using vibrational spectroscopy. SERS represents a highly sensitive method and can be performed using latent fingerprints, but will need to be scaled for the patient bedside [93,94]. As such, traditional Raman and FTIR spectroscopy are ideal methods for sweat analysis and promising diagnostic tools.

**Figure 4:**
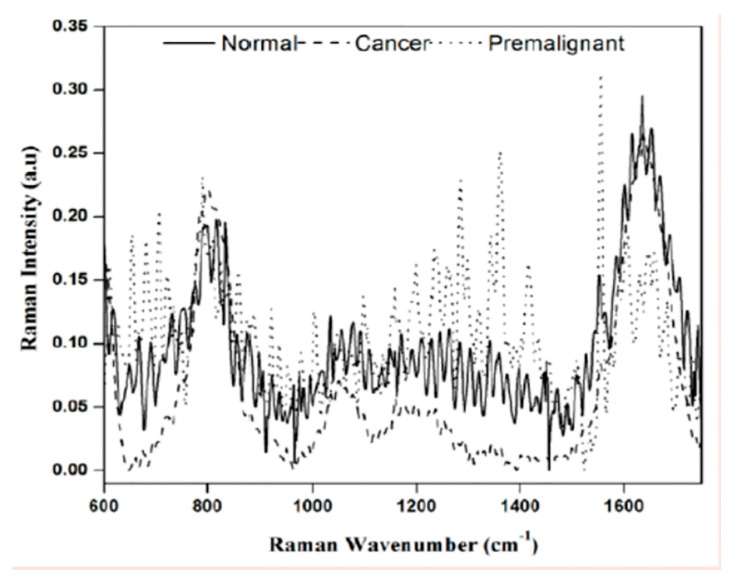
Raman spectroscopic intensity variations and peak shift among saliva samples from three groups [85].

Saliva: Saliva is a bodily fluid that is primarily composed of water, proteins and other minerals. It is produced by secretory glands primarily present in the oral cavity [95]. The ease of salivary collection coupled with the proximity of this biofluid to oral and respiratory tissues has made it a popular candidate fluid for the analyses of periodontal disease, sickle cell anemia, cystic fibrosis and other hereditary disorders, systemic and autoimmune diseases, oral cancers, dental caries and viral infections including novel Covid-19, as evidenced in 2019 pandemic [96,97,98]. Fourier Transform Infrared (FTIR) spectroscopy coupled with Raman spectroscopy have both been utilized as biomarker identifiers in salivary samples [97]. Analysis from the saliva exosome reveals differences in amino acids, carbohydrates, nucleic acids, proteins, and lipids [98,99,100,101,102]. Recently, Paschotto and colleagues have pointed out the importance of saliva collection and processing methods having a major influence on the sensitivity and specificity of the metabolites detected during vibrational spectroscopy. Specifically, the collection and processing have a major effect on the ability to obtain high quality spectra. Thus, unprocessed samples versus samples induced by chewing on cotton with or without centrifugation may differ in quality as well as the particular degree of amino acids, carbohydrates, nucleic acids, proteins, and lipids present in the sample as will degree of drying whether passively or by vacuum centrifugation [103]. Therefore, depending on the specific needs during diagnosis, a particular methodology of collection and processing may be preferred. For carbohydrate-based analyses, supernatant after centrifugation collected by cotton may be a preferred method. For amino acid-based analyses, saliva supernatant that is not caused by cotton saturation may be preferred [103]. Recently, oral squamous cell cancer has been detected in saliva using the SERS enhancement of Raman spectroscopy [104]. Figure 4 shows saliva sample Raman peaks contrasts between normal, cancer and premalignant saliva: for pyrimidine (767, 1236, 1330, 1662 and 1688 cm^−1^ in normal), amide (1652 cm^−1^ normal > malignant > premalignant), mucin 1444 cm^−1^, hemocyanin (752 cm^−1^, sharper for normal saliva—broader for malignant and premalignant), carotenoids (1158 and 1525 cm^−1^, absent in normal).

**Figure 5:**
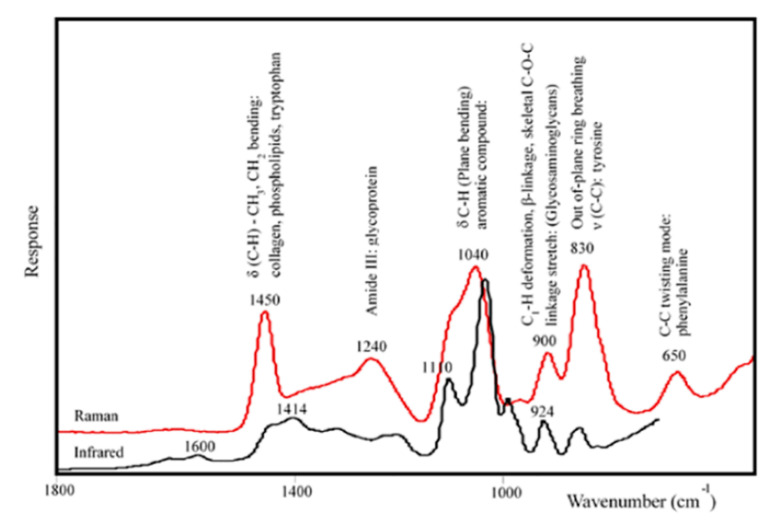
Representative spectra from serum of a subject with Fibromyalgia collected by FT-Raman (red line) and FT-IR (black line) microspectroscopy [39].

Blood: When biofluids are discussed, blood is usually one of the first fluids that come to mind. Blood is composed of various cell types, sugars, electrolytes, organic species, and proteins [85,105]. Despite the collection method of this biofluid being more invasive than some of the other biofluids that are discussed, vibrational spectroscopy in blood is well documented and the fluid can contain diverse biomarkers of disease and damage [3,85]. In fact, the fluid has already been used in the diagnosis of adenocarcinoma, HIV/AIDS, and diabetes [105,106]. Researchers recently used Raman and FTIR spectroscopy techniques to compare blood serum samples between healthy individuals and individuals with depression. This study identified no significant differences between dried and liquid serum samples suggesting that blood remains a viable sample after drying [107]. Further, a significant decrease in peak intensity was realized in functional groups associated with phospholipids and proteins in the depressed patients. Another study using FTIR and Raman spectroscopy compared blood plasma from patients with Alzheimer’s disease (AD) to plasma from non-demented controls [108]. These techniques allowed for highly accurate discrimination between the study groups via metabolites associated with inflammation, oxidative stress, and lipid metabolism. Finally, SERS analysis of liquid blood has proven to be a targeted, inexpensive, and sensitive diagnostic method [109]. SERS analysis of blood can be employed in a rapid, nondestructive, and label-free manner to identify information about various analytes. Contrarily, SERS can also allow for the selective identification and restriction of the target molecule to a specific area when specific labels conjugate to their target ligand. Owing to blood’s diverse makeup, the specificity and selectivity of vibrational spectroscopy will allow physicians to analyze minute concentrations of unique biomarkers that may correlate to damage or diseases in patients [108] Figure 5 shows a representative FT-Raman (red line) and FT-IR (black line) microspectroscopic spectrum from serum of a patient with Fibromyalgia. These spectra highlight the complementary nature of the techniques. The FT-IR spectrum is dominated by the strong vibration modes of water (OH stretching mode centered at 3400 cm^−1^), glucose (C-OH stretching at 1040 cm^−1^), polysaccharides (CO and CC ring vibration at 1110 cm^−1^), lipids (CH_3_ and CH_2_ stretching bands at 2940 and 2880 cm^−1^), and a weak signal in the 1450–1200 cm^−1^ range associated with proteins, phosphate-carrying compounds, and lipids. In contrast, the FT-Raman spectrum showed major bands centered at 650, 830, 900, 1040, 1240 and 1445 cm^−1^ associated with vibrations of aromatic amino acids groups, glycans, collagen, and mineral content of samples (Figure 5). Additionally, while intravenous blood collection appears to be the most reliable collection method for this biofluid, vibrational spectroscopy of blood has proven to be flexible to the unique diagnostic needs and/or the preferred collection method of the healthcare practitioners [3,85,104,105,106,107,108].

**Figure 6:**
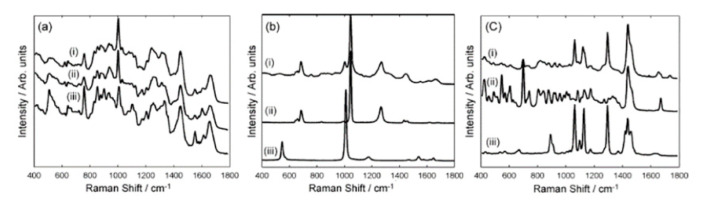
Comparison of tear spectra to biochemical standards, (**a**) proteins, (i) tear sample, (ii) human lactoferrin, (iii) human lysozyme, (**b**) metabolites, (i) tear sample, (ii) sodium bicarbonate, (iii) urea, and (**c**) lipids, (i) tear sample, (ii) cholesterol, (iii) palmitic acid [115].

Tears: Tears are a biofluid composed of various proteins, lysozyme, lipids, urea, and mucins [110]. It is produced in the lacrimal glands and can be easily collected from the conjunctival sac [111]. Since tear fluid can be easily collected, it has been considered as a potential biofluid for medical diagnostics; however, the only diagnostic studies we identified to date was a SERS study that utilized the urea concentration in tears to assist in the diagnosis of renal diseases [112]. Additional studies suggest the potential of vibrational spectroscopy on this biofluid for the diagnosis of ocular disease and infection, but further studies are needed [111,113,114]. Recent FTIR analysis of tears identified phosphate esters, phospholipids, and phosphoproteins that varied significantly between right and left eyes of subjects [115] Figure 6a shows Raman spectra from the edge of a dried tear pattern, and standard chemicals sodium bicarbonate and urea wavelengths shown in Figure 6b. These metabolites (bicarbonate and urea) are commonly found in relatively high concentrations (2 and 0.3 mg·mL^−1^, respectively) in tear fluid. The signals from these two components match up well with the two sharp peaks seen in the loadings of Figure 6a,c, confirming that they are concentrated in, and especially at the edges, of the dried tear patterns (Figure 6). Another FTIR study identified various tear lipids and meibum that could be reliably partitioned using this technique, which may allow researchers to address dry eye issues [3]. Drop Coating Deposition Raman Spectroscopy (DCDRS) of tear fluid has also been conducted and unique spectra of protein groups were created following mass separation [116,117]. There are various methods that allow for non-specific and specific analyses of tear fluid as a biofluid; however, further studies may elucidate the diagnostic value of tears. 

**Figure 7:**
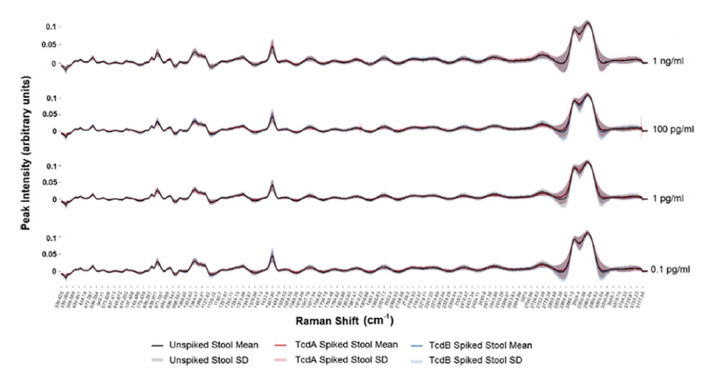
Mean Raman Spectra: Mean Raman spectra for unspiked stool, TcdA-spiked stool and TcdB-spiked stool with respective standard deviation for 1 ng/mL (top), 100 pg/mL (second from top), 1 pg/mL (third from top) and 0.1 pg/mL (bottom) were plotted on *x*-axis for Raman shift 300–3200/cm and their intensities in arbitrary units on *y*-axis [119].

Fecal Material: Fecal material is composed of various cells, lipids, indigestible carbohydrates and is created as a waste product through the process of digestion as food product progresses through the digestive tract [118]. Fecal material expels naturally; therefore, collection is quick, minimally invasive, and can be performed by the patient privately, rather than by a healthcare team member. To date, this biofluid has been used to diagnose steatorrhea, *Clostridium difficile* (CD) infections, gastrointestinal disorders, and various other diseases using fecal fat [118,119,120]. FTIR analysis of fecal fat was compared to the traditional collection method of fecal fat as a diagnostic method for steatorrhea [119]. The study identified FTIR as a rapid, reliable, and simple technique for diagnosing and monitoring steatorrhea with adequate precision compared to the traditional method. Further analysis of fecal material with Near-Infrared Reflectance Spectroscopy (NIRS) demonstrated the ability to reveal the chemical composition of feces [120]. Recently, Koya et al. prepared stool samples with CD toxin A (TxA) and CD toxin B (TxB) to assess the specificity and sensitivity of Raman spectroscopy in stool compared to the traditional CD testing technique [119]. The researchers found Raman to be moderately to highly sensitive with low to moderate specificity in this study. Figure 7 shows the mean Raman spectra of the unspiked, TcdA spiked, and TcdB spiked stool at all concentrations (1 ng/mL, 100 pg/mL, 1 pg/mL and 0.1 pg/mL). Overall, there are promising results for FTIR, NIRS, and Raman spectroscopy as diagnostic tools for stool as a biofluid. These analysis techniques allow for rapid, cost-efficient methods for analyzing samples that typically took a significant amount of time. Additionally, this method can be performed without extraneous reagents or sample preparation.

**Figure 8:**
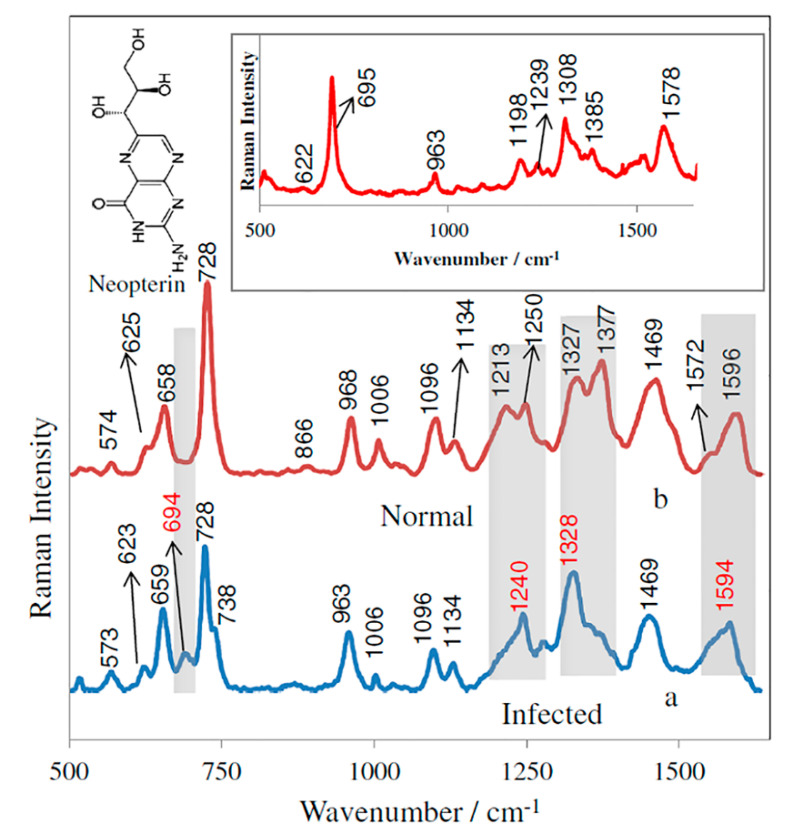
Comparison of the SERS spectrum of the CSF samples infected by N. meningitidis (**a**) versus that of the normal (control) CSF samples (**b**). Samples of CSF were deposited onto the Si/ZnO/Au substrate and measured in situ. The inset shows the SERS spectrum of neopterin adsorbed onto the Si/ZnO/Au substrate from 35.0 nmol/L neopterin solution in a PBS buffer. The presented SERS spectra were averaged from ten measurements in different places of the SERS nanostructures [126].

Cerebrospinal Fluid: Cerebrospinal fluid (CSF) is composed of a unique concentration of sodium and potassium, sparse cells, various proteins, enzymes, and other substances [121]. This biofluid is produced via plasma filtration and membrane filtration and composition can vary according to plasma compositions and various disease states. While CSF is collected through generally invasive means, such as lumbar puncture [121,122], the fluid has proven useful for the diagnosis of inflammatory conditions, infections, and noninfectious diseases of the brain, spinal cord, and meninges [122,123]. Historically, CSF changes are useful in identifying disease states and whether that disease might be inflammatory, metabolic, etc.; however, CSF has not been as useful in providing an exact diagnosis [121]. FTIR analysis of CSF from AD patients and healthy controls identified increased levels of tau protein and decreased levels of beta-Amyloid protein in AD patients compared to the control patients [122,123]. Specifically, this technique was inexpensive, simple, and resulted in high sensitivity and specificity. Researchers recently constructed an artificial CSF to assess the ability of broad infrared spectroscopy to reliably detect concentration variations and other differences in various metabolites, specifically metabolites that are known biomarkers of neurodegenerative disorders, such as albumin and lactate [124]. Infrared spectroscopy identified exclusive spectral fingerprints for albumin and lactate that can distinguish these metabolites, while assessing whether the concentrations of these two metabolites are normal or abnormal. Raman spectroscopy was recently performed on ex vivo CSF from subjects who were *Myobacterium tuberculosis* positive to assess specificity and sensitivity of tuberculosis meningitis [125]. This technique utilized silicate Raman peaks to achieve a 91% sensitivity and 82% specificity, comparable to the traditional testing method. Using these various vibrational spectroscopy techniques on CSF allow for comparable diagnostic accuracy, decreased test cost, elimination of dyes and reagents, and a dramatically improved time to diagnosis. SERS has been used to enhance the detection capabilities in cases of Neisseria meningitis [126]. Figure 8 shows the normalized SERS spectra of CSF samples infected by N. meningitidis (a) and the normal (control) CSF samples (b) deposited onto the Si/ZnO/Au substrate. Aromatic amino acid residues, phenylalanine, tyrosine, and tryptophan were expected to have bands at 625, 659, 866, 1173, and 1213 cm^−1^. The prominent SERS peaks located at 659, 728, 963, 1006, 1096, 1134, and 1469 cm^−1^ can also be consistently observed in infected samples (Figure 8b). However, a detailed analysis of SERS spectra of CSF from patients affected by bacterial meningitis reveals a new SERS band at 695 cm^−1^ which corresponds to the C–C vibration and ring modes of neopterin can be observed only in the infected samples and is absent in healthy subjects. This indicates the increased contribution of neopterin in bacterial infections (Figure 8).

**Figure 9:**
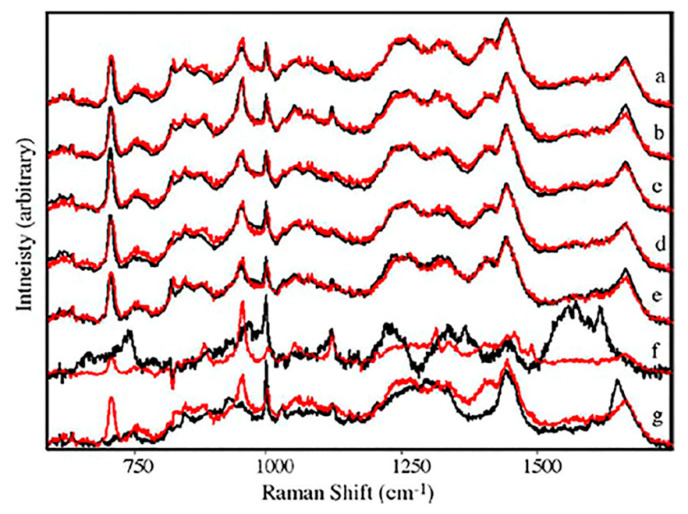
The average Raman spectra of five semen samples (black) with the fitted spectroscopic signature (a–e), and the Raman spectra of blood (f) and saliva (g) with the fitted spectroscopic signature [128].

Semen: Semen is a reproductive fluid comprised of sperm (reproductive component), carbohydrates, proteins, electrolytes, and various other constituents that can be collected non-invasively [127,128,129]. This biofluid has been used in the past for the diagnosis of male reproductive disorders [108] and bacterial prostatitis [130,131]. FTIR analysis of semen revealed unique lipids, protein and phosphate peaks that could be uniquely assigned to semen, rather than other biofluids [131,132,133]. Additional FTIR analysis elucidated cells, seminal plasma, amino acids, citrate, enzymes, and several other components that could be separated based on unique spectral identities [132]. These studies suggest that FTIR is a reliable technique for diagnosis and analysis of semen relative to conventional methods, while being less expensive, easy to use, and more comprehensive. Raman analysis of semen revealed unique spectral fingerprints in semen compared to other fluids, such as amides, amino acids and spermine phosphate hexahydrate [133,134,135]. Further Raman analysis enabled the separation of semen samples from blood, sweat, saliva, and vaginal fluid samples [133] A spectroscopic signature was created that consisted of the three principal components found in the basis semen sample. The signature was also applied to the spectra from each of 49 remaining semen samples. Figure 9 shows the fitting to only five representative semen samples, but all of the samples had very similar fits. The bottom of Figure 9 contains the results of fitting the spectroscopic signature to the spectra of blood and saliva, and it is obvious that they are very poor matches. These data signatures show the potential ability to be used as an identification technique for forensic purposes (Figure 9). Of note, choline, fructose, tyrosine, spermine phosphate hexahydrate, lactate, and other metabolites were found to have unique spectral outputs in semen compared to other specimens. Raman analysis also enabled the identification of unique amino acid peaks in UV damaged semen compared to normal semen [134]. The ability to diagnose seminal DNA integrity using Raman spectroscopy has significant value in the diagnosis and prognosis of male urological disorders [129,134,135]. As such, multiple vibrational spectroscopy modes have shown success in the diagnosis of related diseases and in revealing the various components of semen; therefore, this method is promising for the identification of biomarkers in semen that will allow for the early detection of damage and disease states using this biofluid.

**Figure 10:**
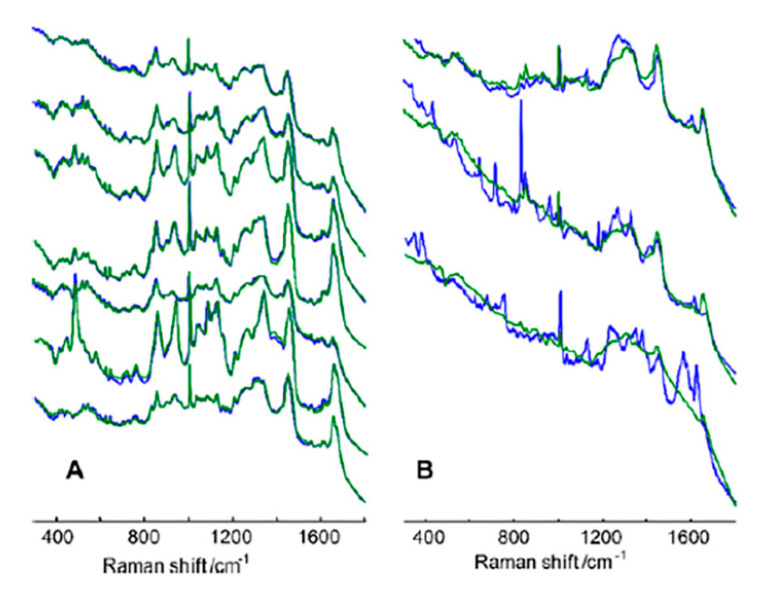
The average Raman spectra of seven vaginal fluid samples (**A**) and the Raman spectra of saliva, semen and blood (**B**, top, middle and bottom lines, respectively) with the fitted spectroscopic signature of vaginal fluid [136].

Vaginal Fluid: Vaginal fluid is typically a mucus or secretion composed of bacteria, fungi, carbohydrates, cells, proteins, and various other components [136]; however, vaginal fluid can also exist as an abnormal discharge due to various disease states [137,138]. This biofluid has been used for the diagnosis of vaginitis [137], bacterial infections [137,138,139], and inflammatory diseases [139]. FTIR analysis of vaginal secretions identified unique spectral peaks corresponding to vaginal fluid carbohydrates, amides, and epithelia [140]. Raman spectroscopic techniques were performed on vaginal fluid, which allowed for the simple, fast, and nondestructive identification of significantly different spectral peaks between this biofluid and other biofluids [136,137]. FTIR comparison of menstrual blood, vaginal fluid, and seminal fluid allowed for 100% discrimination in an inclusive, rapid, reliable, and non-destructive manner based on spectra corresponding to phosphoric acid, amide groups, and methyl groups [140]. FTIR was also used to analyze cells that are frequently found in vaginal fluids [140]. This study identified multiple variables in the sample and suggests that neural network discrimination and/or multivariate statistical analyses would allow for the identification of bacterial infections, early signs of cancer, and human papilloma virus. Raman spectroscopy of vaginal fluid was performed to assess separation techniques between sample types and to determine if Raman techniques would render the sample nonviable [141]. Vaginal fluid was successfully and reliably separated from semen, sweat, saliva, and blood based on spectral fingerprints An analysis hard-constrained to the spectral profiles of the complete vaginal fluid signature (three fluorescent components, three Raman components (Figure 10A,B) and a horizontal line) was successfully used to fit the averaged spectra found from the remaining vaginal fluid samples (Figure 10). Blue lines represent the experimental spectra, while green lines are the result of fitting. The diversity of the experimental spectra was evident, but the multidimensional signature was able to include all of the key characterizing moieties (Figure 10). Furthermore, unique spectra were identified for urea, lactate, acetic acid, and other metabolites in vaginal fluid. Recent studies have explored the role of Interleukin-1α (IL) as a vaginal fluid biomarker in endometriosis [141]. This study reported high specificity (100%) and sensitivity (100%) of IL in the diagnosis of endometriosis. Raman spectroscopy recently allowed for the reliable and sensitive identification of interleukin-6 [142], suggesting that this technique can successfully detect cytokine presence. Vibrational spectroscopy techniques may allow for easy, comprehensive diagnostics of vaginal fluid, while eliminating the need for additional resources, reagents, or expensive machines.

**Figure 11:**
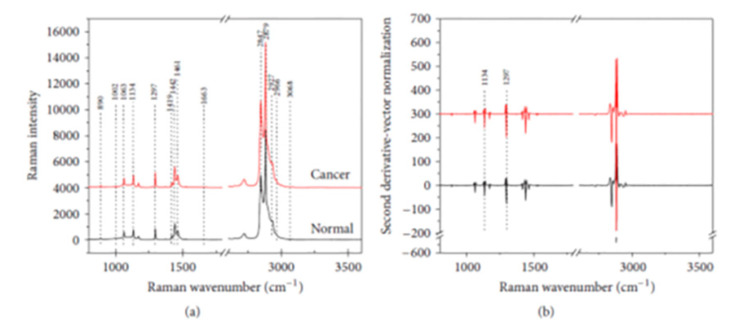
(**a**) Averaged Raman spectra for normal (*n* = 78) and cancerous (*n* = 81) colorectal tissues. (**b**) Second derivative-vector normalized spectra of Raman spectra from normal and cancerous tissues. The broken interval (-//-) indicates the region of 1800–2600 cm^−1^, which does not contain tissue-related biochemical information [151].

Tissue Samples: Tissues can be found throughout the body and can be composed of a diverse and unique set of components based on where these tissues present in the body [143]. Specifically, tissue exists of cells, an extracellular matrix made with various proteins (e.g., collagen and elastin), and a base layer derived from proteoglycans; however, the exact makeup of this tissue can vary significantly. These samples are typically collected via surgical biopsies [144]; however, laparoscopy, bronchoscopy, and other minimally invasive techniques have been employed recently [144,145,146]. Tissue is used extensively and is well researched as a diagnostic sample for various diseases, such as osteoarthritis, cancer, osteoporosis, and intervertebral disk degeneration [146,147,148]. FTIR spectroscopy recently allowed for rapid, simple, and reliable histopathologic recognition independent of pathologist interpretation and absent visual morphology via unique spectral outputs [149]. Furthermore, FTIR spectroscopy compared colon samples with diagnosed colon cancer or without colon cancer to assess for spectral variation that would allow for confident separation of sample groups [150,151,152]. The spectral results noted particularly decreased hypophosphate peaks associated with nucleic acids in the colon cancer samples, which allowed for the differentiation of these groups with 100% sensitivity (Figure 11). Prominent Raman bands were observed for normal and cancerous colorectal tissue at about 1063 (lipids/collagen), 1134 (fatty acids and proteins), 1174 (Ltryptophan), 1297 (lipids and phospholipids), 1414 (lipids), 1442 (fatty acids and triglycerides), 1461 (lipids/proteins), 2847 (fatty acids and triglycerides), 2879 (lipids), and 2927 cm^−1^ (proteins and lipids). Peak intensities at 1134 and 1297 cm^−1^ increased significantly in cancerous tissue, relative to the normal tissue, suggesting a higher amount of lipid material compared with the normal tissue. Various Raman spectroscopy techniques, such as SERS and transmission Raman spectroscopy, were performed on human tissue samples from diverse anatomical regions. Spectral variance in urea, cholesterol, proteins, and other components allowed for diagnosis of breast cancer, lung cancer, skin cancer, esophageal diseases, prostate cancer, colorectal cancer, and several other diseases with sensitivity and specificity comparable to traditional diagnostic techniques [14,151]. Recently, Sinica et al. utilized Raman techniques to compare metabolites between cancerous and non-cancerous lung tissue [152]. The team identified unique Raman results for glycogen, aromatic amino acids, phospholipids, and other metabolites associated with anaerobic metabolism and damage. The spectral differences were able to distinguish normal lung tissue from abnormal lung tissue (cancer or other lung damage) with an original accuracy of 96% (84% after cross validation). Based on these results, vibrational techniques for the identification of varying metabolite concentration has been well documented in a diverse range of tissues. Furthermore, multiple vibrational techniques allow for sample processing according to the unique needs of the healthcare team.

## 2. Conclusions

We herein discuss the use of current vibrational techniques for the identification of metabolite presence and concentration differences. Infrared spectroscopy of tissues, cells, and various biofluids allows for the elucidation of the organization and classification of lipids, carbohydrates, proteins, and metabolites [5,12,16]. In turn, the unique composition and vibrational spectra of chemicals in these human samples have demonstrated the potential of IR spectroscopy in the diagnosis of a range of diseases or damage states. Raman spectroscopy has demonstrated viability in non-invasive and in vivo diagnostics, while further advancements may allow for integrated Raman devices that could be easily adopted as wearable devices or in the clinical setting [105,132,149]. Miniaturization of vibrational spectrometers into commercially available portable and handheld Raman and infrared (IR) spectrometers has occurred within the last few years, driven by developments in micro-electro-mechanical systems (MEMS) production. Portable/handheld optical systems for chemical identification have incorporated the analytical precision of spectroscopy to field applications with spectral resolution equivalent to bench-top instruments. Thus, the only barrier for the further utilization of vibrational spectroscopy in the clinics is the further development and fine tuning of the MEMS currently being developed. The current gold standard for analyses of biofluids discussed are primarily NMR spectroscopy and mass spectrometry (MS) techniques, which provide selectivity and specificity for screening metabolites but require costly instrumentation, involve labor-intensive and complex sample pretreatment, and require well-trained technicians to operate the instrumentation thus being less amenable to point of care delivery. Overall, these techniques allow for the early, accurate, quick, inexpensive, and easy diagnosis of diseases. Specifically, metabolites were found in all of the biofluids discussed above that allowed for successful and reliable differentiation of samples and between healthy and non-healthy samples. These findings support the broad use of vibrational spectroscopy as a diagnostic tool due to increasing portability, ease of use, low cost, and reliability. Further vibrational research may elucidate specific biomarkers in biofluids that can be utilized for the diagnosis of other diseases and abnormal states. The various methods of vibrational spectroscopy can be utilized in a standalone method, collectively, or in different groupings based on specific needs or according to research backing to allow for confirmatory diagnosis of diseases using a variety of biofluids and sample types.

## Figures and Tables

**Table 1:** Overview of clinical studies performed by using Raman spectroscopy as a diagnosis tool in vivo in humans.

Methodology	Disease Type/Metabolite	Patient Number	Results			Reference
			Sensitivity	Specificity	Accuracy	
Probe (830 nm)	Nondysplastic Barrett’s esophagus (NDBE)	62	86	88		[20]
Probe (785 nm)		65	86	88	87	[21]
Confocal Probe (785 nm)	Barrett’s esophagus (BE)	373	87	84.7		[22]
Probe (785 nm)	Cervical Cancer	63	100	96.7		[23]
Probe (785 nm)		93				[24]
Confocal Microscope (532 nm)		30	100	100	100	[25]
Portable (785 nm)	Cervical Precancer	172			97	[26]
Portable with probe (785 nm)		79	89	81		[27]
Portable with probe (785 nm)		145			94	[28]
Benchtop with probe (785 nm)	Lung Cancer	10	94	92		[29]
Portable with probe (785 nm)	Brain Cancer	17	93	91		[30]
Portable with probe (785 nm)		10	84	89	87	[31]
Microscope	Osteoarthritis	40	74	71		[32]
Probe (830 nm)	Skin Cancer	76	100	100	100	[33]
Probe (785 nm)		104	74	82		[34]
Confocal Probe (785 nm)	Oral Cancer	84	92.7 (tumor)	98.66 (control)		[35]
Confocal Microscope (785 nm)	Oral Cancer (urine tested)	167	98.6	87.1	93.7	[36]
Dispersive (830 nm)	Prostate Cancer (blood tested)	107	87.41	76.47		[37]
Confocal Microscope (785 nm)	Atopic Dermatitis	132	98.73	86.89		[38]
Confocal Microscope (1064 nm)	FM *, RA *, SLE *	88	no misclassified sample			[39]

* FM: Fibromyalgia, RA: Rheumatoid Arthritis, SLE: Systemic Lupus Erythematosus.

**Table 2:** Overview of clinical studies performed by using FT-IR spectroscopy as a diagnosis tool in vivo in humans.

Methodology	Disease Type/Metabolite	Patient Number	Results			Reference
			Sensitivity	Specificity	Accuracy	
Benchtop ATR	Breast Cancer	100	90	98	94	[40]
Miscroscope Trans-reflectance		20				[41]
Benchtop Transmission		86	76	72	80	[42]
Benchtop Trans-reflectance		196	Cluster analysis: 98	Cluster analysis: 95		[43]
			ANN *: 92	ANN: 100		
Microscope	Breast Cancer-ECM3 * gene	97	91	95		[44]
Benchtop ATR–FTIR	Skin Cancer	7				[45]
Benchtop	Cervical Precancer		100	95.2	97.4	[46]
Benchtop	Leukemia	34	83.3	79		[47]
Microscope		51				[48]
Microscope ATR	Colon Cancer	78	100	93.1	95.8	[49]
Benchtop ATR	Ovarian Cancer (blood tested)	30	100	100		[50]
Benchtop	Gastric Cancer (serum tested)	46	100	80		[51]
Microscope	Lung Cancer		99.3	94.4	96.8	[52]
Microscope Trans-reflectance	Lung Tumor (tissue tested)	112			97	[53]
Benchtop Transmittance	Bladder Cancer	136	100	58.8		[54]
Benchtop Reflectance			90	80.9		
Benchtop Transmission	Malignant Biliary Strictures	57	90	100		[55]
Benchtop	Invasive Ductal Carcinoma	229	91.7	100	95.7	[56]
Benchtop ATR	FM *	252	89.5	79	84.2	[57]
Microscope ATR	FM, OA *, RA *	41			100(SIMCA *); 75(RF *)	[58]
Portable ATR	FM, RA, SLE *	70	no misclassified sample			[39]
Benchtop	Burning Mouth Syndrome (saliva tested)	28	Area under the ROC curve calculated as 0.75			[59]
Microscope	Human Papillomavirus	50	76.9	76.7		[60]

* ANN: Artificial Neural Networks, ECM3 gene: Extracellular Matrix Cluster 3, SIMCA: Soft Independent Modeling Class Analogy, RF: Random Forest, FM: Fibromyalgia, OA: Osteoarthritis, RA: Rheumatoid Arthritis, SLE: Systemic Lupus Erythematosus.

**Table 3:** Overview of the analytes detected from biofluids using Raman and FT-IR spectroscopy.

Methodology	Analyte	Tested Biofluid	Limit of Detection	Reference
Dispersive Raman 785 nm—SERS	Cocaine	Saliva	25 ng/mL	[61]
FT-Raman 785 nm—SERS	5-Fluorouracil		150 ng/mL	[62]
Handheld Raman 633 nm—SERS	S100P mRNA *		1.1 nM	[63]
FT-Raman 785 nm—SERS	Cocaine, PCP *, diazepam, acetaminophen		Cocaine: 50 ng/mL, PCP: 1 mcg/mL, diazepam: 1 mcg/mL, acetaminophen: 10 mcg/mL	[64]
Raman Microscope 633 nm—SERS	Morphine		2.4 × 10^−4^ ng/mL	[65]
Raman Microscope 633 nm—SERRS *	Hemozoin - Malaria Biomarker	Blood	30 parasites/μl	[66]
Raman Microscope 633 nm—SERS	d-Glucose		1 µM	[67]
Raman—SERS	*E. coli*, *S. aureus*, *P. aeruginosa*		*E. coli*: 3 × 10^4^ CFU/mL, *S. aureus*: 3 × 10^3^ CFU/mL, *P. aeruginosa*: 5 × 10^3^ CFU/mL	[68]
Raman Microscope 633 nm—SERS	Interleukins (IL-6, IL-8, IL-16)		IL-6: 2.3 pg/mL, IL-8: 6.5 pg/mL, IL-16: 4.2 pg/mL	[69]
Raman Microscope 785 nm	Red blood cell		250 fL (as little as a single red blood cell)	[70]
Raman Microscope 514 nm—IERS *	TAFC-Biomarker of invasive aspergillosis	Urine	0.5 ng/mL	[71]
Raman—S-SERS *	Creatinine		0.68 mg/dl	[72]
Raman Microscope 785 nm—SERS	Mephedrone, nor-mephedrone, 4-methylephedrine		~2 nM (0.41 g/L)	[73]
Handheld Raman 785 nm—SERS	Methamphetamine (MA), 3,4-methylenedioxymethamphetamine (MDMA), and methcathinone (MC)		0.1 ppm	[74]
FT-IR benchtop	Candida, *Gardnerella vaginalis*	Vaginal fluid	Candida: 0.25 × 10^2^ CFU/mL, *Gardnerella vaginalis* 1 × 10^2^ CFU/mL	[75]
FT-IR benchtop	Thiocyanate	Saliva	140 pM	[76]
FT-IR benchtop—ATR *	Cocaine		10 µg/mL	[77]
FT-IR	CYFRA-21-1 biomarker of oral cancer		0.122 ng/mL	[78]
FT-IR benchtop—single reflection ATR	Albumin	Urine	6.7 ppm	[79]
FT-IR benchtop—nine reflection ATR	Lidocaine		0.5 mg/L	[80]
FT-IR benchtop—ATR	Synthetic cannabinoids		JWH-018: 0.3 pg/mL, JWH-073: 0.45 pg/mL, JWH-018 pentanoic acid: 0.4 pg/mL, JWH-073 butanoic acid: 0.2 pg/mL	[81]

* S100P mRNA: S100 calcium-binding protein P mRNA—a potential biomarker for oral cancer, PCP: (1-(1-phenylcyclohexyl) piperidine), SERRS: surface-enhanced resonance Raman spectroscopy, IERS: interference-enhanced Raman spectroscopy, TAFC: Triacetylfusarinine C, S-SERS: stamping surface enhanced Raman scattering, ATR: Attenuated total reflectance.
